# A Scoping Review of Factor-Related Cognitive Impairment in Interstitial Lung Disease

**DOI:** 10.7759/cureus.67791

**Published:** 2024-08-26

**Authors:** Hiroki Annaka, Kenta Honma, Tomonori Nomura

**Affiliations:** 1 Department of Occupational Therapy, Faculty of Rehabilitation, Niigata University of Health and Welfare, Niigata, JPN

**Keywords:** quality of life, review, carbon monoxide pulmonary diffusing capacity, pulmonary rehabilitation, cognitive impairment, interstitial lung disease

## Abstract

Understanding the risk factors for cognitive impairment in interstitial lung disease (ILD) can help guide disease management tailored to cognitive function. However, no review articles or randomized controlled trial articles have been found for cognitive impairment in ILD. This scoping review aimed to systematically map studies on factor-related cognitive impairment in ILD and organize current knowledge. Literature on cognitive impairment in ILD was retrieved from PubMed, Scopus, and EBSCO and manually searched using Google. Three researchers screened the relevant literature. Six studies were extracted: four were case-control studies; one was a cross-sectional study; and one was a prospective cohort study. The extracted literature lacked studies with a high level of evidence and only reported factor-related cognitive impairment in ILD, not risk factors. Factors related to cognitive impairment were carbon monoxide pulmonary diffusing capacity, FEV1/FVC, hospitalization for lung transplantation, delirium during hospitalization, apnea-hypopnea index and Epworth sleepiness scale scores, idiopathic pulmonary fibrosis, abnormal pulmonary artery pressure, hypoxemia, post-exercise arterial blood oxygen partial pressure and heart rate, and six-minute walk test results. This scoping review presents the current knowledge on the risk factors for cognitive dysfunction in ILD. The extracted literature did not include reports on the risk factors for cognitive impairment in ILD and was limited to reports on related factors. Building evidence on this topic is desirable for understanding the risk factors for cognitive impairment in patients with ILD.

## Introduction and background

Interstitial lung disease (ILD) is a general term for lung diseases that cause scarring and fibrosis of lung tissue. The clinical course and prognosis of this group of diseases vary greatly depending on the disease type. Particularly, idiopathic pulmonary fibrosis (IPF), which is an ILD with progressive pulmonary fibrosis, progresses rapidly and has a poor prognosis, with a survival of approximately three years [1]. The number of deaths from ILD in Japan is increasing, and in 2022, ILD was the ninth leading cause of death among men according to Japan's vital statistics [2]. Although no radical treatment has yet been established for ILD, the ATS/ERS/JRS/ALAT clinical practice guidelines for IPF and progressive pulmonary fibrosis indicate that therapeutic management should include antifibrotic medications, oxygen therapy, and pulmonary rehabilitation [3]. The primary therapeutic goal for ILD is to continue therapeutic management to slow disease progression and prolong survival [4].

The Global Initiative for Chronic Obstructive Lung Disease notes the negative impact of cognitive impairment on the therapeutic management of chronic obstructive pulmonary disease (COPD) [5]. Cognitive impairment decreases adherence to medications and oxygen therapy, leading to acute exacerbations [6,7]. Besides affecting treatment management, this impairment prevents patients from living independent daily lives, resulting in the loss of self-esteem and reduction in the quality of life (QOL) [8]. The more severe the cognitive impairment, the shorter the survival time in patients with chronic respiratory diseases [9]. Early detection of cognitive impairment and management tailored to cognitive function are necessary to maintain QOL and prolong healthy life expectancy in pulmonary rehabilitation [7,8].

The prevalence of cognitive impairment in chronic respiratory disease varies depending on the study methodology or the type and severity of the disease being studied; however, it can be as high as 70% in severe cases [9], and review articles on COPD have reported on the risk factors for cognitive impairment in chronic respiratory disease [10-12]. Chronic hypoxemia associated with pulmonary dysfunction is a significant risk factor because it reduces oxygen levels in the brain and causes cranial nerve damage [10,11]. Systemic inflammation associated with the disease causes microvascular damage in the brain, leading to cranial nerve damage [10,11]. Smoking, obstructive sleep apnea, and hospitalization for acute exacerbations are also risk factors [10]. Understanding these risk factors will help in the early detection of cognitive impairment in patients and contribute to the management of cognitive impairment in pulmonary rehabilitation [13].

Therapeutic management of ILD is critical for maintaining QOL and prolonging survival, and the inability to continue treatment due to cognitive impairment leads to fatal outcomes in patients with ILD. Therefore, cognitive impairment should be identified and managed early. Review articles have reported on the risk factors for cognitive impairment in COPD; however, no review articles or randomized controlled trial articles were found for those in ILD. The purpose of this scoping review was to systematically map the literature on factor-related cognitive impairment in ILD and organize current knowledge. This review will help professionals involved in pulmonary rehabilitation understand the current knowledge on factor-related cognitive impairment in ILD.

## Review

Materials and methods

This scoping review was based on the Preferred Reporting Systems for Systematic Reviews and Meta-Analyses Extension for Scoping Reviews (PRISMA-ScR) framework [14]. Table 1 summarizes the framework used in this study.

**Table 1:** Framework for this study

Research question	
Population	Interstitial lung disease
Concept	Factor-related cognitive impairment
Context	The included study is unaffected by study location, culture, or race. The criteria were "research article," "English language," and "research on cognitive impairment in ILD"

The study criteria were "research article," "English language," and "research on cognitive impairment in ILD." Literature searches were conducted using PubMed, Scopus, and EBSCO databases as well as by a hand search using Google, with no limitation on the publication period. One researcher (H. A.) conducted the search on February 4, 2024. Table 2 lists the search strategy used in this study. The limits in PubMed were set to "English" for language, "human" for subject, and "full text" for availability.

**Table 2:** The literature search strategy

Database	Search strategy
PubMed and Scopus	(“Interstitial pneumonia”OR“Interstitial lung disease”OR “Lung Diseases, Interstitial[MeSH]”)AND (“cognition[Mesh]”OR“cognitive function”OR “cognitive impairment”OR“cognitive dysfunction”)
EBSCO	“Interstitial pneumonia or Interstitial lung disease”AND“cognitive impairment or cognitive dysfunction”

Literature selection and eligibility check were performed by two researchers (H. A. and K. H.) using the Rayyan screening system [15] and independently reviewed against the criteria. The studies were validated for the title and abstract in the first stage of screening and for the main text in the second stage. When the two researchers disagreed, the eligibility of the study was reviewed by a third researcher (T. N.).

Data were extracted from the literature by a single researcher (H. A.). Data extracted included "year of publication," "title," "country," "subject," "design," and "finding."

Results

**Figure 1:**
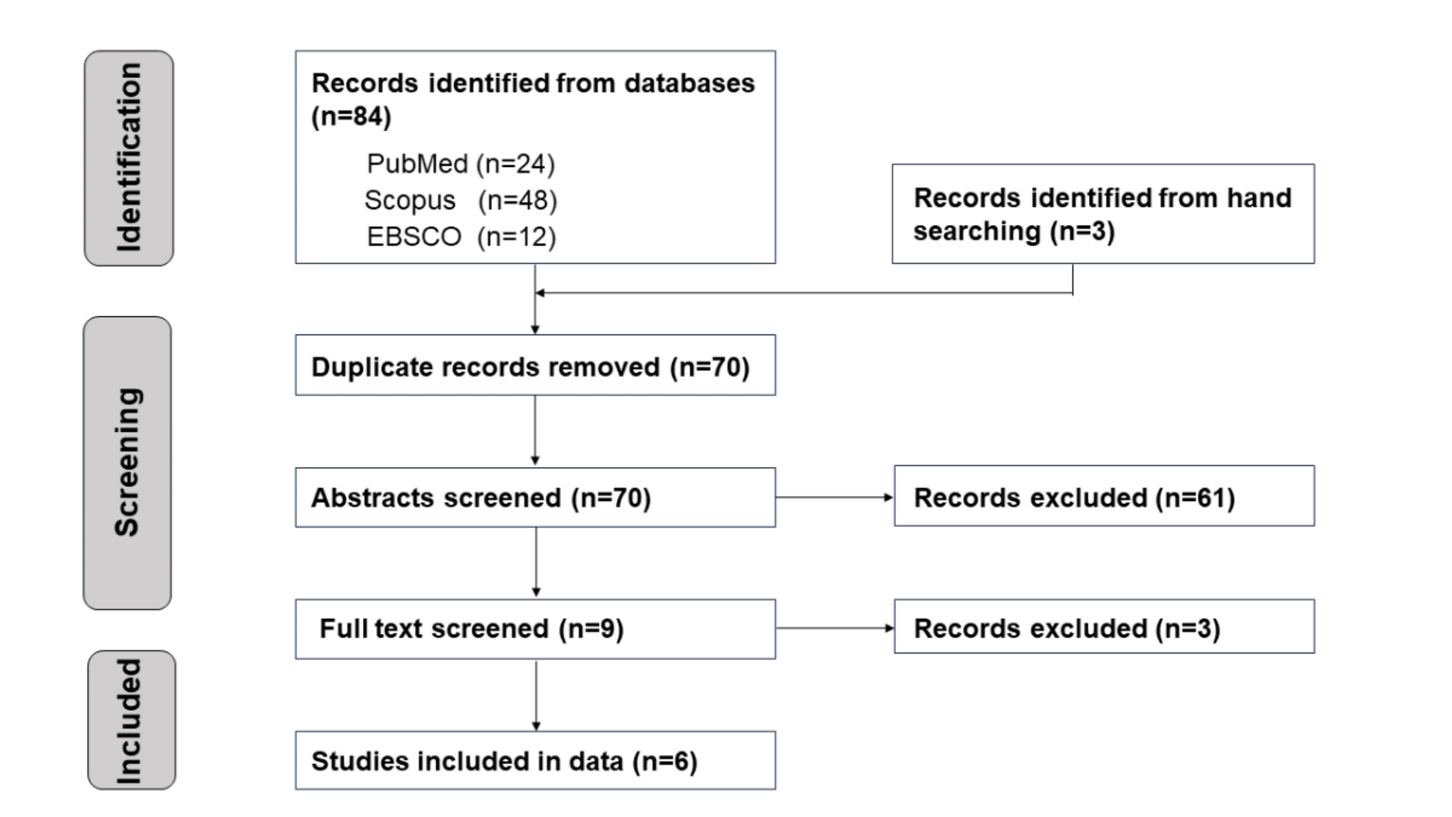
Flowchart of study selection

A flowchart of the literature selection process is shown in Figure 1.

Eighty-four articles were found from the following databases: 24 from PubMed; 48, Scopus; and 12, EBSCO. The hand search retrieved three studies. Of the total, 14 studies were excluded because of duplication, resulting in 70 studies. In the first stage of screening, nine studies were extracted. Of the 61 excluded studies, three were excluded following a discussion with a third researcher. Those excluded papers were mistakenly included by one researcher. In the second stage of screening, six studies were considered eligible for this review [16-21]. A summary of the six studies is presented in Table 3. These included four case-control studies [16,18-20], one prospective cohort study [17], and one cross-sectional study [21].

**Table 3:** Summary of study characteristics ILD: interstitial lung disease; IPF: idiopathic pulmonary fibrosis; COPD: chronic obstructive pulmonary disease; DLco: diffusion capacity of lung carbon monoxide

No.	Year	Title	Country	Subject	Design	Finding
1	2014	Cognitive function in idiopathic pulmonary fibrosis [16]	USA	Severe IPF (n=12). Mild-to-moderate IPF (n=34). Control (n=15)	Case-control study	Among patients with IPF, those with DLco <30% had worse cognitive function compared to those with DLco >30%
2	2014	Neurocognitive changes after lung transplantation [17]	USA	Lung transplant IPF (n=17). COPD (n=8). Cystic fibrosis (n=8). Others (n=14)	Prospective cohort study	IPF, COPD, and other diseases caused transient cognitive decline after lung transplantation that improved three months after discharge. Seven patients with IPF (41%) had delirium after lung transplantation. The median duration of delirium was two days. Patients with delirium showed cognitive decline three months after discharge
3	2019	Impact of moderate to severe obstructive sleep apnea on the cognition in idiopathic pulmonary fibrosis [18]	Italy	Moderate-to-severe IPF (n=23). COPD (n=30). Control (n=17)	Case-control study	Patients with IPF with a Montreal Cognitive Assessment score of <23 points had higher apnea-hypopnea index and Epworth sleepiness scale scores compared to those with IPF with a Montreal Cognitive Assessment score of ≥23 points
4	2020	Study of cognitive functions in major idiopathic interstitial pneumonias [19]	Egypt	Severe ILD (n=30). Control (n=20)	Case-control study	Patients with ILD had lower cognitive function than controls. Patients with IPF had lower cognitive function than patients with other ILD
5	2021	Cognitive impairment and depression in patients with idiopathic pulmonary fibrosis [20]	Egypt	IPF (n=100). Control (n=100)	Case-control study	Patients with IPF with abnormal pulmonary artery pressure had lower cognitive function than those with IPF with normal pulmonary artery pressure. Patients with IPF with severe hypoxemia had lower cognitive function than those with IPF with mild hypoxemia
6	2022	Neuropsychological functioning in patients with interstitial lung disease [21]	Greece	ILD (n=51). Control (n=88)	Cross-sectional study	Cognitive function in ILD was associated with post-exercise partial pressure of oxygen, post-exercise heart rate, six-minute walk test result, DLco, and FEV1/FVC

Patient Characteristics

Of the six studies, two and four studies involved patients with ILD and IPF, respectively. Three studies were conducted in patients with severe disease [16,18-19]. The severity classification differed among the three studies; it was based on carbon monoxide pulmonary diffusing capacity (DLco), forced vital capacity, and the gender-age-physiology (GAP) index. Smith et al. [17] reported on IPF, COPD, and cystic fibrosis after lung transplantation.

Factor-Related Cognitive Impairment

Six studies reported factor-related cognitive impairment in ILD.

Two studies reported the relationship between lung function and cognitive impairment. Bors et al. [16] found that cognitive function was worse in patients with IPF with DLco <30% than that in patients with IPF with DLco >30. Giannouli et al. [21] showed that cognitive function in ILD is related to DLco and FEV1/FVC.

Two studies reported the relationship between hypoxemia and cognitive impairment. One study compared cognitive function between patients with IPF with severe hypoxemia and those with mild hypoxemia [20], and another study examined the relationship between cognitive function and post-exercise arterial partial pressure of oxygen in ILD [21], which showed that cognitive function was related to severe hypoxemia.

The relationship between ILD type and cognitive impairment was shown by Zakaria et al. [19], with IPF being associated with worse cognitive function than other ILD.

The relationship between cardiovascular efficiency, such as post-exercise arterial oxygen pressure, post-exercise heart rate, and six-minute walk test results, was associated with cognitive function [21].

The relationship between comorbidities and cognitive impairment was reported in two studies. IPF with abnormal pulmonary artery pressure [20] and IPF with a high apnea-hypopnea index and Epworth sleepiness scale score worsen cognitive function.

Studies on the impact of lung transplantation have shown cognitive decline at hospital discharge after lung transplantation, but improvement at the three-month follow-up [17]. In contrast, patients with IPF who developed delirium during hospitalization did not show improved cognitive function at the three-month follow-up.

Discussion

The study systematically mapped the literature on factor-related cognitive impairment in ILD according to the PRISMA-ScR framework and identified six studies: four case-control studies, one cross-sectional study, and one prospective cohort study. The extracted literature lacked studies with a high level of evidence and only reported factor-related cognitive impairment in ILD. We attribute this finding to the fact that risk factors for cognitive impairment are viewed in the same way in ILD as in COPD. However, this scoping review suggested factor-related cognitive impairment specific to ILD from the limited literature.

Factor-Related Cognitive Impairment in ILD

This study identified factors associated with cognitive impairment in ILD from six studies; these included DLco, FEV1/FVC, lung transplantation, delirium during hospitalization, apnea-hypopnea index and Epworth sleepiness scale score, IPF, abnormal pulmonary artery pressure, hypoxemia, post-exercise arterial blood oxygen partial pressure and heart rate, and six-minute walk test results.

Reviews on COPD reported decreased FEV1/FVC, apnea-hypopnea index and Epworth sleepiness scale scores, abnormal pulmonary artery pressure, hypoxemia, and six-minute walk test results as risk factors for cognitive impairment [10]. These factors reduce oxygen levels in the brain and cause cranial nerve damage [10].

However, to our knowledge, DLco, IPF, and post-exercise arterial partial pressure of oxygen and heart rate have not been reported in COPD and may be factors specific to ILD. DLco is a measure of gas exchange between the alveoli and capillaries and is reduced in the early stages of ILD, which is characterized by fibrosis. It is also used as an indicator of ILD severity, like the GAP index [22]. Although a decreased FEV1/FVC ratio and forced vital capacity have been reported as risk factors for cognitive impairment in chronic respiratory diseases, a decrease in DLco may also be a risk factor for ILD. In addition, a decrease in the post-exercise arterial partial pressure of oxygen and an increase in the post-exercise heart rate are characteristic symptoms of ILD. In COPD, resting arterial partial pressure of oxygen has been shown to be a risk factor for cognitive impairment [10]. In ILD characterized by a decrease in DLco, hypoxemia occurs after exercise even in the absence of hypoxemia at rest. Capturing the post-exercise arterial partial pressure of oxygen and heart rate in ILD may be useful for the early detection of cognitive impairment due to decreased oxygen supply to the brain.

IPF is an ILD with progressive pulmonary fibrosis, and the inflammatory response and hypoxemia associated with fibrosis are more pronounced than those in other ILD [3]. The pathophysiology of IPF may cause cognitive decline due to more extensive cranial nerve damage than that in other types of ILD. Future studies are required to examine how different types of ILD affect cognitive function.

Hospitalization for lung transplantation and delirium during hospitalization were associated with cognitive impairment. However, cognitive impairment may be related to various factors, such as age, length of hospitalization, and degree of surgical invasiveness; therefore, further investigation is needed. In Japan, the number of lung transplants is increasing every year, with ILD accounting for the largest proportion of these transplants [23]. We believe that elucidating the relationship between lung transplantation and cognitive impairment is important for improving patients' QOL.

Limitation

This study had some limitations. First, we included only English-language studies. Second, the five identified studies were cross-sectional, and the six identified studies had small sample sizes; thus, it was not possible to determine a causal relationship between the extracted factors and cognitive impairment. 

## Conclusions

This study summarizes the current knowledge on factor-related cognitive impairment in ILD through a scoping review. We indicated factor-related cognitive impairment in ILD from six studies; these included DLco, FEV1/FVC, lung transplantation, delirium during hospitalization, apnea-hypopnea index and Epworth sleepiness scale score, IPF, abnormal pulmonary artery pressure, hypoxemia, post-exercise arterial blood oxygen partial pressure and heart rate, and six-minute walk test results.

Knowledge of cognitive impairment in ILD is limited. To our knowledge, there are no studies with a high level of evidence on risk factors for cognitive impairment in ILD. Building evidence on this topic is desirable for understanding cognitive impairment in ILD.
